# IL-1β suppresses cLTP-induced surface expression of GluA1 and actin polymerization via ceramide-mediated Src activation

**DOI:** 10.1186/s12974-018-1158-9

**Published:** 2018-04-30

**Authors:** Liqi Tong, G. Aleph Prieto, Carl W. Cotman

**Affiliations:** 1 Institute for Memory Impairments and Neurological Disorders, University of California, 1226 Gillespie Neuroscience Research Facility, Irvine, CA 92697 USA; 2 Department of Neurobiology and Behavior, University of California, 1226 Gillespie Neuroscience Research Facility, Irvine, CA 92697 USA

**Keywords:** IL-1β, Synaptic plasticity, Dendritic spine, GluA1, Actin dynamics

## Abstract

**Background:**

Brain inflammation including increases in inflammatory cytokines such as IL-1β is widely believed to contribute to the pathophysiology of Alzheimer’s disease. Although IL-1β-induced impairments in long-term potentiation (LTP) in acute hippocampal slices and memory functions in vivo have been well documented, the neuron-specific molecular mechanisms of IL-1β-mediated impairments of LTP and memory remain unclear.

**Methods:**

This study uses an in vitro approach in primary hippocampal neurons to evaluate the effect of IL-1β on chemical LTP (cLTP)-induced structural plasticity and signaling.

**Results:**

We found that IL-1β reduces both the surface expression of alpha-amino-3-hydroxy-5-methyl-4-isoxazolepropionic acid (AMPA) receptor subunit GluA1 and the spine growth following cLTP. These effects of IL-1β were mediated by impairing actin polymerization during cLTP, as IL-1β decreased the cLTP-induced formation of F-actin, and the effect of IL-1β on cLTP-induced surface expression of GluA1 can be mimicked by latrunculin, a toxin that disrupts dynamics of actin filaments, and can be prevented by jasplakinolide, a cell-permeable peptide that stabilizes F-actin. Moreover, live-cell imaging demonstrated that IL-1β decreased the stability of the actin cytoskeleton in spines, which is required for LTP consolidation. We further examined the role of sphingolipid signaling in the IL-1β-mediated impairment of spine plasticity and found that both the neutral sphingomyelinase inhibitor GW4869 and the inhibitor of Src kinase PP2 attenuated the IL-1β-mediated suppression of cLTP-induced surface expression of GluA1 and actin polymerization.

**Conclusions:**

These findings support a mechanism by which IL-1β, via the sphingomyelinase/ceramide/Src pathway, impairs structural spine remodeling essential for LTP consolidation and memory.

## Background

Brain inflammation is widely believed to underlie the pathophysiology of Alzheimer’s disease (AD) [1–4]. The pro-inflammatory cytokine IL-1β plays a pivotal role in brain inflammation and mediates the effect of inflammation on cognition and synaptic plasticity. Accumulating evidence indicates that elevated IL-1β levels cause cognitive decline, especially on hippocampal-dependent tasks [5]. IL-1β overexpression in an inducible transgenic mouse shows impaired hippocampal-dependent long-term contextual and spatial memory with normal non-hippocampal memory [6]. In addition, peripheral *Escherichia coli* infection increases IL-1β levels in the hippocampus and impairs contextual fear conditioning in old animals, and the loss of memory is prevented by the specific IL-1β receptor antagonist IL-1ra [7, 8]. Several reports have demonstrated that IL-1β suppresses long-term potentiation (LTP) [9–11], a form of synaptic plasticity broadly defined as an activity-dependent increase in synaptic strength and considered to be a cellular mechanism for memory formation. In an aged animal, inflammation impairs theta-burst-induced LTP (theta-LTP), which is thought to mimic the firing patterns observed in the hippocampus during behavioral learning in animals in vivo [12], via IL-1β signaling [13]. In transgenic mouse models of AD, the deficit in LTP correlates with increased expression of IL-1β [14, 15]. Blocking IL-1 signaling by IL-1 receptor antibody prevented amyloid-beta (Aβ)-induced impairment on LTP and memory [16].

Dendritic spines are a major site of information processing in the brain. Therefore, mechanisms that regulate the plasticity of dendritic spines are fundamental to cognitive functions including learning and memory (for review, see [17]). It is well known that activity-driven changes in synaptic efficacy modulate spine morphology due to alterations in the underlying actin cytoskeleton. Excitatory synaptic transmission is tightly regulated by the total number and activation of AMPA receptors (AMPARs) present at the synapse. During LTP, AMPARs are inserted into the postsynaptic membrane, the spine heads enlarge, and synaptic connections are strengthened [18, 19]. Actin polymerization has been proposed to be a critical event for the stabilization of LTP [18, 20]. Thus, actin dynamics plays an important role in synaptic development and plasticity.

Our previous study has shown that IL-1β impairs the stabilization of theta-LTP and actin polymerization in acute hippocampal slices [21]. Whether IL-1β suppresses activity-dependent actin polymerization by direct effects on neurons or by indirect mechanisms mediated by non-neuronal cells is poorly understood. Elucidating neuron-specific effects of IL-1β in hippocampal slices has been challenging because most brain cells express IL-1 receptors and IL-1β increases the expression of multiple cytokines in their target cells. Here, to study neuron-specific effects and further focus on dendritic spines, we used chemical LTP (cLTP) in low-density primary neuronal cultures, an experimental system commonly used to elucidate molecular and cellular changes during LTP [22]. The characterization of glycine-induced cLTP has confirmed that the induction method shares many key properties with theta-LTP of CA1 neurons [23]. For example, LTP in dissociated hippocampal neuronal cultures is also dependent on Ca^2+^ influx through post-synaptic NMDA receptors, subsequent activation and autophosphorylation of the Ca^2+^/calmodulin-dependent protein kinase II (CaMKII), and an increase in AMPAR receptor insertion at the post-synaptic membrane [24, 25].

Here, we show that IL-1β impairs cLTP-induced structural spine remodeling underlying plasticity, including surface insertion of GluA1-containing AMPAR, spine enlargement, and actin polymerization in primary cultured hippocampal neurons. We also investigated the possible role of sphingolipid signaling in the IL-1β-mediated suppression of cLTP.

## Methods

### Hippocampal cell culture and transfection

The use of all animals was approved by the Institutional Animal Care and Use Committee at the University of California, Irvine. Primary hippocampal cell cultures were previously described [26]. Briefly, primary hippocampal neurons were obtained from the hippocampi of Sprague–Dawley rat embryos (E18-19) (Charles River). Hippocampi were incubated in Hank’s Balanced Salt Solution (HBSS; Invitrogen, USA) with 0.025% trypsin (Invitrogen) for 10 min at 37 °C. Neurons dissociated in NeuroBasal medium (Invitrogen) supplemented with B27 (Invitrogen), and penicillin/streptomycin were plated on poly-D-lysine (Sigma)-coated six-well plates for Western blot analysis and glass bottom dishes (Mattek) for immunocytochemistry. The neuronal cultures were maintained in Neurobasal medium supplemented with 2% B27 supplement, 0.5% GlutaMax, and 1% penicillin/streptomycin mix (Invitrogen) in a 37 °C, 5% CO_2_ incubator. The neurons were transfected using lipofectamine 2000 (Invitrogen) following the supplier’s protocol.

### DNA constructs, reagents, and treatments

tdTomato and Lifeact-GFP were purchased from ibidi, Inc. Ser3 peptides with the sequence of MAS(p)GVAVSDGVIKVFN were synthesized by GenScript (GenScript). GW4869, desipramine, fumonisin B1, C2-ceramide, and Pyrazolopyrimidine 2 (PP2) were purchased from Sigma-Aldrich (Sigma-Aldrich, USA). Recombinant IL-1β (PeproTech) was dissolved in DMEM and used after one freeze–thaw cycle at 50 ng/ml. After 14–18 days in vitro (DIV), neurons were treated at 37 °C with 50 ng/ml IL-1β, with control neurons receiving equal volumes of vehicle. cLTP was induced as described previously [24, 25]. Briefly, hippocampal neurons were maintained in normal ACSF (5 mM HEPES [pH 7.3], 125 mM NaCl, 2.5 mM KCl, 2 mM CaCl2, 1 mM MgCl2, and 33 mM glucose). Osmolarity was adjusted to 290 mosmol/l. Chemical LTP was induced by changing the medium to Mg^2+^-free ACSF (5 mM HEPES [pH 7.3], 125 mM NaCl, 2.5 mM KCl, 2 mM CaCl2, 33 mM glucose, 0.2 mM glycine, 0.02 mM bicuculline, and 0.003 mM strychnine) for 10 min. After that, the incubation solution was altered back to control solution without glycine for 20 min before surface GluA1 labeling and for 30 min before fixation for immunohistochemistry to detect changes in F-actin, respectively. Neurons were treated with vehicle or IL-1β before (1 h), during (10 min), and after cLTP stimulation at indicated time points.

### Western blotting

Cultures were washed twice with cold PBS prior to incubation in RIPA buffer (Thermo Fisher) containing a proteinase and phosphatase cocktail (Thermo Fisher) for 20 min on ice. Lysates were centrifuged at 12,000×*g* at 4 °C for 20 min. The total amount of protein in cultures was determined using BCA protein quantitation assay (Pierce), according to the manufacturer’s protocol. Equivalent amounts of protein for each sample were electrophoresed on 4–12% SDS-polyacrylamide gel (Lonza). Proteins were then electrotransferred to PVDF membranes (Bio-Rad), blocked with 5% nonfat milk in Tris-buffered saline (TBS), and probed with various antibodies. The membrane was stained with an appropriate primary antibody overnight at 4 °C. The following antibodies were used as indicated: phosphorylated Src (P-Src, detects Src phosphorylated at Tyr416, 1:1000, Cell signaling), Total-Src (T-Src, 1:1000, Millipore), total cofilin (T-Cof, 1:1000, Millipore), phosphorylated cofilin (P-Cof, detects cofilin phosphorylated at Ser3, 1:2000, Millipore), and actin (1:2000, Cytoskeleton). The immunoreactivity was revealed using horseradish peroxidase-conjugated secondary antibody (goat anti-rabbit IgG or goat anti-mouse IgG, Vector Laboratories) and SuperSignal Wester Dura Extended Duration Chemiluminescence Substrate (Thermo Fisher) according to the recommended conditions. The membranes were developed using AFP Imaging Developing System (MRX) or Bio-Rad ChemiDoc Imaging System (Bio-Rad), respectively. Immunoreactivity was quantified using ImageJ (NIH). Quantification of the data obtained from Western blots derived from cultures under the various experimental conditions was analyzed.

### Assay for the ratio of G-actin to F-actin

The ratio of G-actin to F-actin was analyzed using a kit (Cytoskeleton) according to manufacturer’s instructions. Briefly, after treatment, cells were lysed in a detergent-based lysis buffer that stabilizes and maintains the G– and F– forms of cellular actin. Cell lysates were transferred to an ultracentrifuge and spun at 150,000×*g* for 1 h to separate the globular (G)-actin (supernatant) and filamentous (F)-actin fractions (Beckman). Samples of supernatant and pellet were electrophoresed on SDS-polyacrylamide gel. All samples were diluted with appropriate loading buffer and boiled for 5 min. Actin was quantified by Western blot analysis.

### FRAP (fluorescence recovery after photobleaching)

The 14–16 DIV neurons were transfected with Lifeact-GFP. For the imaging, the cell culture dishes were placed on an imaging stage in the microscope environmental chamber (37 °C, 5% CO_2_). FRAP experiments were performed using a macro function of the stimulus setting menu in LSM510 software to control sequential image acquisition and emission of a photobleaching laser pulse to the ROI (region of interest). A single dendritic spine of hippocampal neuron was set as ROI and five pre-bleaching images acquired at 5-s intervals and the fluorescence of spine photobleached with an Argon 488 laser at low power (2–4%) to avoid photobleaching during the time-lapse imaging; the emitted light was passed through a band pass emission filter. The recovery of fluorescence was traced for an additional 5 min by acquiring images at 5-s intervals. Minimum laser power was used to prevent photobleaching during the pre- and post-bleaching stages. Pre-bleaching, bleaching, and post-bleaching images were utilized for analyzing the dynamics of target proteins. Images were taken by a confocal laser scanning microscope (CLSM) (Zeiss LSM 510) and quantified with ImageJ. The mobile fraction (f_m_)  and the immobile fraction (fi) were calculated by the following equations: f_m_ = F_∞_/ F_0_, where F_∞_ is the fluorescence intensity after full recovery, and F_0_ is the fluorescence intensity before photobleaching, f_i_ = 1 − f_m_.

### Immunocytochemistry, confocal imaging, and analysis

Cultured cells were fixed with 4% paraformaldehyde (PFA) and permeabilized using 0.1% TX-100, and non-specific binding of the antibodies was blocked by incubation with 5% normal goat serum (Vector). The permeabilized and blocked samples were incubated with primary antibodies overnight at 4 °C. Excess antibodies were washed off and the samples were incubated with corresponding Alexa Fluor 488 or 555 secondary antibodies for 1 h at room temperature. For surface GluA1 staining, neurons were incubated with an antibody to the N terminus of GluA1 (Calbiochem) in recording solution for 20 min at 37 °C, washed with phosphate-buffered saline (PBS), fixed with 4% paraformaldehyde (PFA, wt/vol) and 4% sucrose (wt/vol) in PBS for 20 min, and incubated with appropriate secondary antibody in 1% BSA (wt/vol) for 45 min before imaging. For F-actin labeling, neurons were first fixed for 20 min in 4% PFA before permeabilizing with 0.1% Triton X-100 (vol/vol) in PBS for 10 min and labeling with Alexa 568–phalloidin for 15 min (Invitrogen, 1:1000). For spine density analysis, dissociated hippocampal cultures were transfected with Lifeact-GFP for 24 h before treatments. After fixation, dendrites were straightened using ImageJ, and spine density was determined by manually counting spines. All immunocytochemistry experiments were performed from at least three individual batches of cultures for different conditions in parallel. Images were acquired by confocal laser scanning microscopy (CLSM) (LSM 510, Zeiss or Olympus Fluoview 3000, Olympus) using identical settings for parallel cultured and quantified using ImageJ. Quantitative analysis of surface GluA1 (sGluA1) expression was carried out in 3D by Z-stacking with 0.5 μm each step for 5–8 steps using a confocal microscope and Volocity software for deconvolution. Quantification of sGluA1 average pixel intensities on the surface of dendrites was carried out using the ImageJ software. Dendrites were identified using threshold adjustment for background fluorescence, and only clearly identifiable dendrites were selected for analysis. All analyses were performed blind to the experimental manipulation. A single value was obtained from each independent experiment and used to construct the mean and standard error. The number of independent experiments was the number of observations used for statistical analysis. For live imaging, neurons expressing Lifeact-mCherry were stimulated by cLTP using glycine stimulation as described above and then imagined by Olympus Fluoview 3000, Olympus) on the stage of 37 °C and CO_2_ environment at the indicated time after glycine stimulation. Images were analyzed by ImageJ.

### Statistical analysis

Data are expressed as means ± SEM and were analyzed using one-way ANOVA followed by a Bonferroni’s post hoc test. The level of significance was set at *p* < 0.05.

## Results

### IL-1β decreased cLTP-induced GluA1surface expression and spine growth

**Fig. 1:**
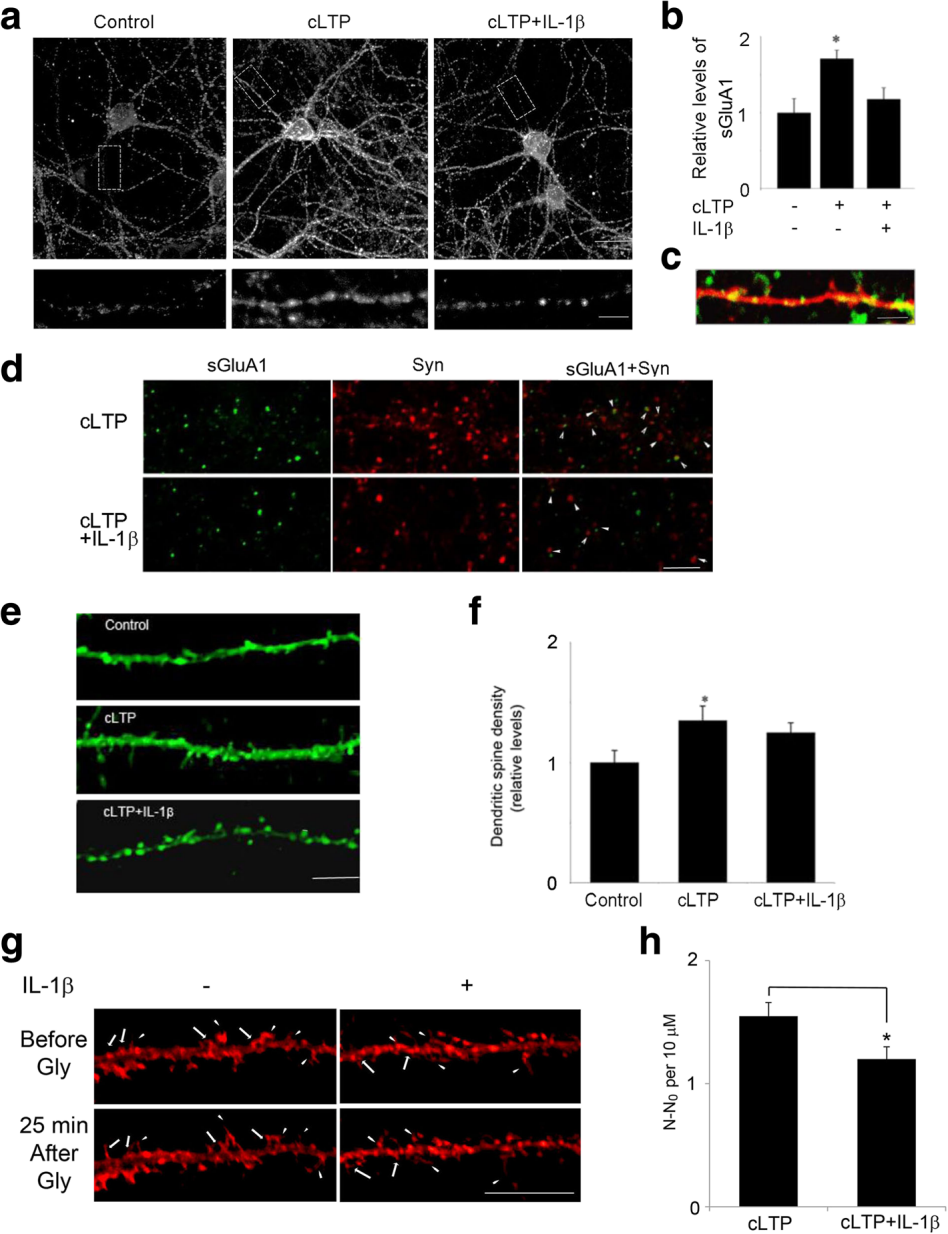
IL-1β impaired cLTP-induced GluA1 insertion and spine formation. **a** Representative fluorescence images show the expression of surface GluA1 (sGluA1) in unstimulated control cells and cells treated with cLTP in the presence or absence of IL-1β. cLTP was induced in hippocampal neurons at 14–18 DIV. sGluA1 level was assessed by immunocytochemistry as described in the “Methods” section. Scale bar: top panel, 10 μm; bottom panel, 5 μm. **b** Quantification of the images shown in **a**. Data are mean ± SEM from three independent experiments expressed in terms of control (*p < 0.05, ANOVA). **c** GluA1 (green) insertion occurs primarily in spines, identified in neurons transfected with TdTomato (red). Scale bar, 5 μm. **d** Cells double-labeled with GluA1, under nonpermeant conditions, and synaptophysin (Syn), under permeant conditions. The co-localization of GluA1 and Syn was illustrated by the arrowhead. Scale bar, 10 μm. **e** IL-1β decreased cLTP-induced growth of dendritic spines. Representative fluorescence images of hippocampal dendritic spines visualized in cells transfected with Lifeact-GFP. Thirty minutes min after cLTP, cells were fixed and image was taken and spine density was quantified. cLTP increased the density of dendritic spines. Scale bar, 10 μm. **f** Quantification of dendritic spine density. Data are mean ± SEM from three independent experiments expressed in terms of spine numbers obtained in the control cultures (*p < 0.05, ANOVA). **g** Glycine stimulation induces new spine formation (arrow) and preexisting spine growth (arrow head). Neurons expressing Lifeact-mCherry were stimulated by glycine (200 μM, 10 min) and then imaged at the indicated time after glycine stimulation. Scale bar, 20 μm. **h** Quantitative of spine formation following glycine stimulation. N number of dendritic protrusions per 10 μm at 25 min after glycine stimulation, N_0_ number of dendritic protrusions per 10 μm before glycine stimulation. Data are mean ± SEM (*n* = 4) (*p < 0.05, Student’s *t* test)

We first tested the possibility that IL-1β impairs GluA1-containing AMPAR trafficking and insertion into spines following cLTP. We used a cell culture model of cLTP in which pharmacological activation of NMDA receptors leads to an increase in the surface expression of synaptic AMPARs [24, 25, 27, 28]. Rat hippocampal neurons (14-18 DIV) were treated with IL-1β, and the surface expression of GluA1 was examined by immunocytochemistry. Consistent with prior results, application of the NMDAR co-agonist glycine in the presence of a GABA receptor antagonist bicuculline led to a significant increase in the surface expression of endogenous GluA1-containing AMPARs compared to unstimulated control cells (Fig. 1a). IL-1β significantly reduced cLTP-induced surface expression of GluA1 (Fig. 1a, b).

Consistent with a previous report [29], GluA1 insertion occurs primarily in spines, identified in neurons transfected with TdTomato (Fig. 1c). To provide direct evidence that glycine-induced LTP promotes GluA1 insertion at postsynaptic sites, we next labeled GluA1, under nonpermeant conditions, and subsequently stained the same neurons for the presynaptic marker protein synaptophysin, under permeant conditions, to identify synapses. Treatment of cultures with glycine increased GluA1/synaptophysin co-localization, whereas IL-1β decreased cLTP-induced GluA1/synaptophysin co-localization (Fig. 1d). These results indicate that IL-1β impairs cLTP-induced increase in the number of GluA1 at postsynaptic sites.

Several studies have demonstrated that LTP increases both the number and size of dendritic spines [25, 29]. To examine the effect of IL-1β on cLTP-induced growth of dendritic spines, we transfected neurons with Lifeact-GFP and measured the formation of new spines in response to cLTP. Consistent with previous reports, the number of dendritic protrusions of existing spines increased after glycine treatment (Fig. 1e, f). cLTP induced a 1.4-fold increase in the total number of dendritic spines. IL-1β treatment decreased cLTP-induced increases in the number of dendritic spines (Fig. 1e, f). We further performed live imaging experiment to test directly whether IL-1β affects cLTP-induced new spine formation. As shown in Fig. 1g, h, cLTP increased new spine formation and IL-1β decreased the effect of cLTP. These results suggest that IL-1β inhibits cLTP-induced structural changes critical to synaptic plasticity.

### IL-1β suppresses cLTP-induced GluA1 surface expression by affecting actin dynamics

**Fig. 2:**
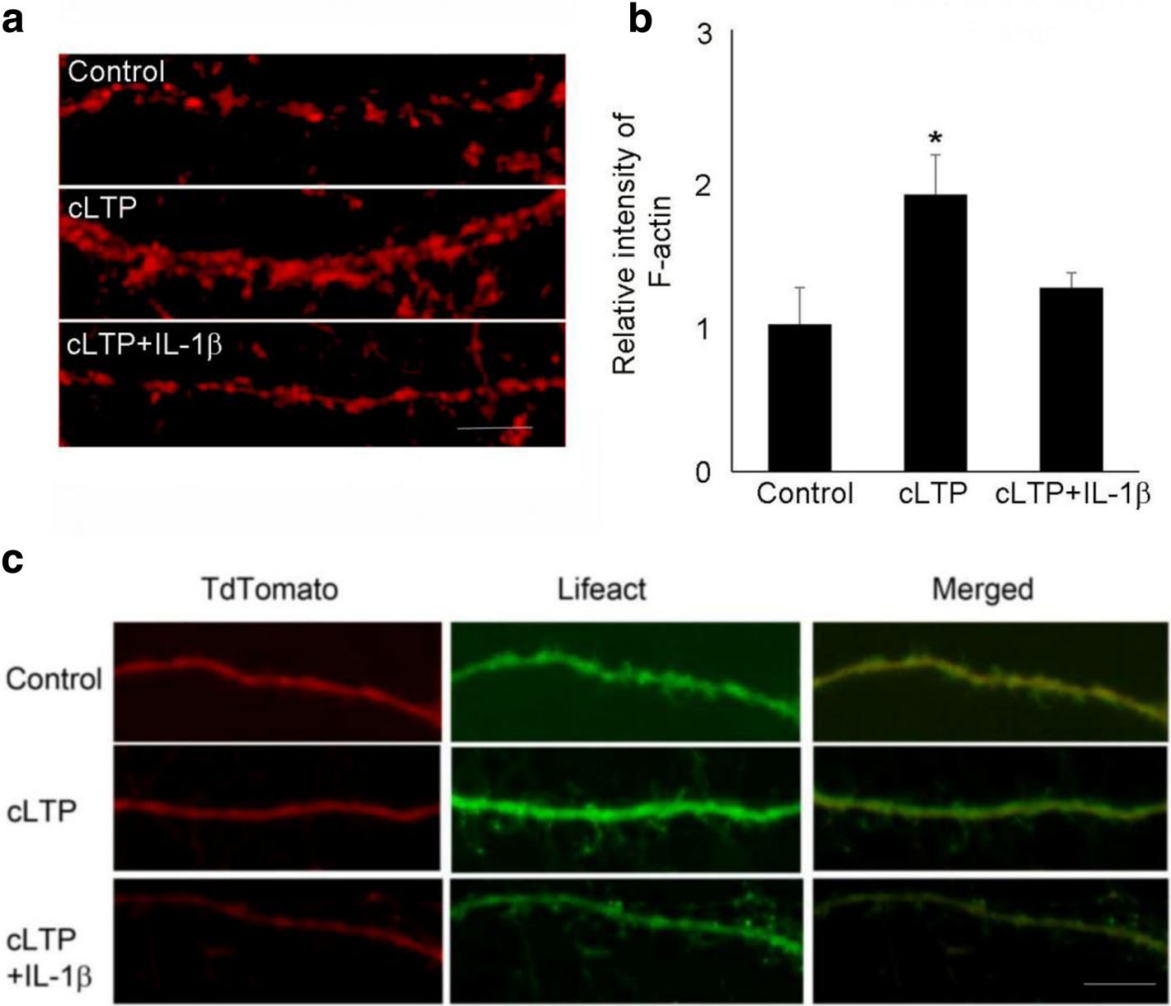
IL-1β impaired cLTP-induced actin polymerization. **a** Cells were fixed following cLTP (30 min) and F-actin staining by phallodin. Scale bar, 5 μm. **b** Quantification of the images shown in **a**. Data are mean ± SEM from three independent experiments expressed in terms of control (*p < 0.05, ANOVA). **c** F-actin in control and cLTP-stimulated cells were labeled by Lifeact/tdtomato co-transfection. Scale bar, 5 μm

LTP at mature excitatory synapses requires both the trafficking of AMPA receptors and the growth of dendritic spines, in which dynamic reorganization of actin cytoskeleton plays a crucial role [30]. There are two forms of actins: monomeric globular actin (G-actin) and polymerized filamentous actin (F-actin). The transition between these two forms is controlled by synaptic activity. We have previously reported that IL-1β decreased TBS-LTP-induced F-actin in acute hippocampal slice [21]. This led us to investigate, in a neuron-enriched experimental system, the effect of IL-1β in both actin polymerization and GluA1 insertion following cLTP. We first examined the effect of IL-1β on cLTP-induced actin polymerization (F-actin), which was measured by phalloidin staining. Consistent with previous reports [25], glycine stimulation increased actin polymerization. We found that treatment of cultured neurons with IL-1β significantly decreased cLTP-induced F-actin (Fig. 2a, b). As an alternative approach, we monitored F-actin with Lifeact, a 17 amino acid peptide which can be attached to a fluorophore and allows visualization of actin dynamics [31]. IL-1β attenuated the induction of F-actin by cLTP, as measured by imaging Lifeact-GFP in neurons co-transfected with tdtomato (to visualize spines) (Fig. 2c).

**Fig. 3:**
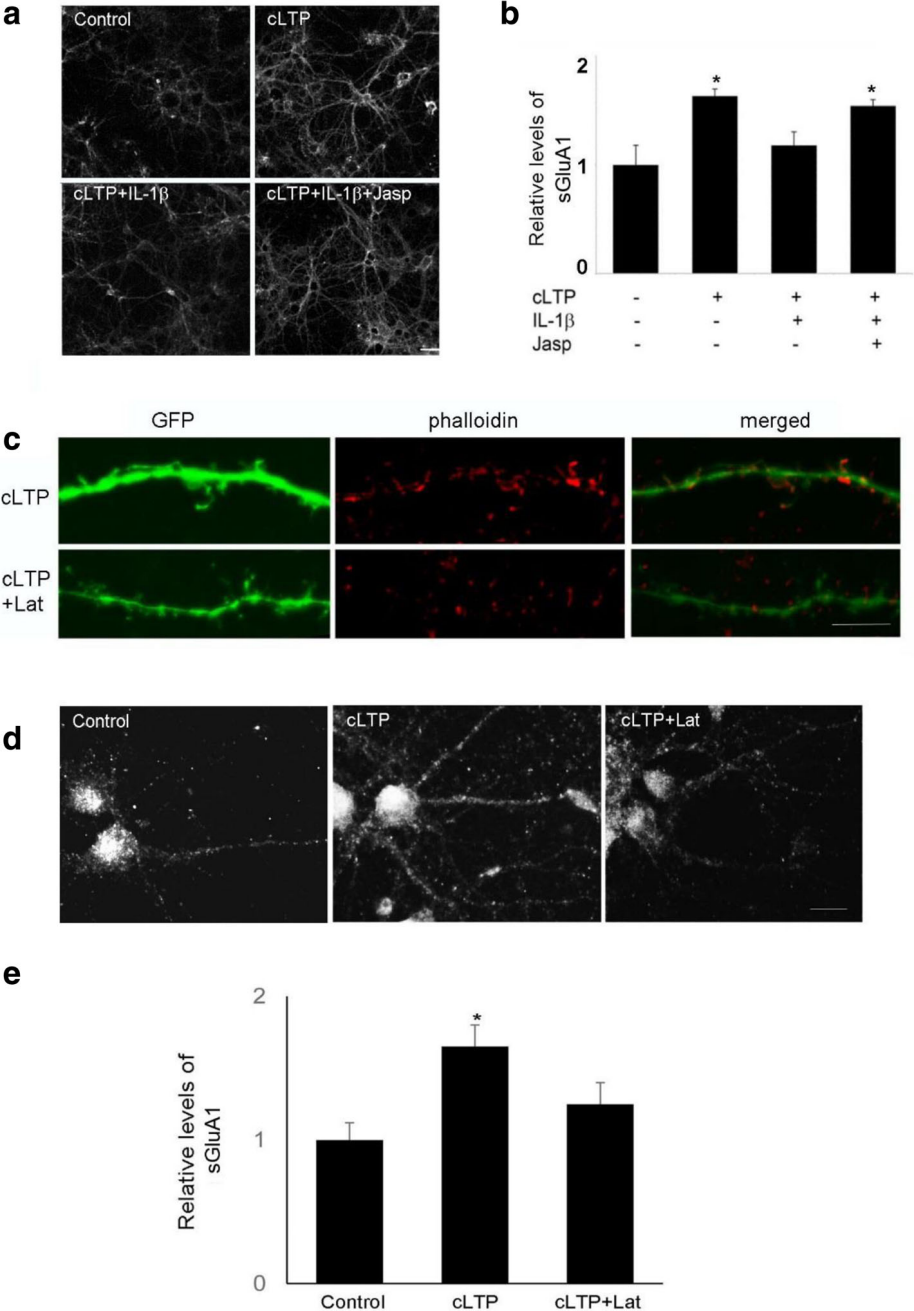
The effect of IL-1β on cLTP-induced GluA1 insertion was mediated by the regulation of actin dynamics. **a** The expression of sGluA1 in unstimulated control cells and cells treated with cLTP in the presence or absence of IL-1β with or without jasplakinolide (200 nM). **b** Quantification of the images shown in **a**. Data are mean ± SEM from three independent experiments expressed in terms of control (*p < 0.05, ANOVA). **c** Cells transfected with GFP were stained by phallodin. **d** The expression of sGluA1 in unstimulated control cells and cells treated with cLTP in the presence or absence of latrunculin A (1 μM). **e** Quantification of the images shown in **d**. Data are mean ± SEM from three independent experiments expressed in terms of control (*p < 0.05, ANOVA)

Because IL-1β-mediated suppression of cLTP-induced GluA1 insertion correlates with decreased actin polymerization, we next tested the possibility that the effect of IL-1β on cLTP-induced GluA1 insertion is mediated by the regulation of actin dynamics. For this purpose, we examined the effect of IL-1β on cLTP-induced surface expression of GluA1 in the presence of jasplakinolide, a cell-permeable peptide that stabilizes actin filaments. Jasplakinolide prevented the inhibitory effect of IL-1β on GluA1 surface expression (Fig. 3a, b). We further examined the effect of latrunculin, a toxin that disrupts dynamic actin filaments, on cLTP-induced surface expression of GluA1. We found that latrunculin decreased cLTP-induced actin polymerization (Fig. 3c) and surface expression of GluA1 (Fig. 3d, e). These results suggest that IL-1β decreases cLTP-induced GluA1 insertion by selectively interfering with actin dynamics.

### IL-1β decreases the stability of the cytoskeleton in dendritic spines

**Fig. 4:**
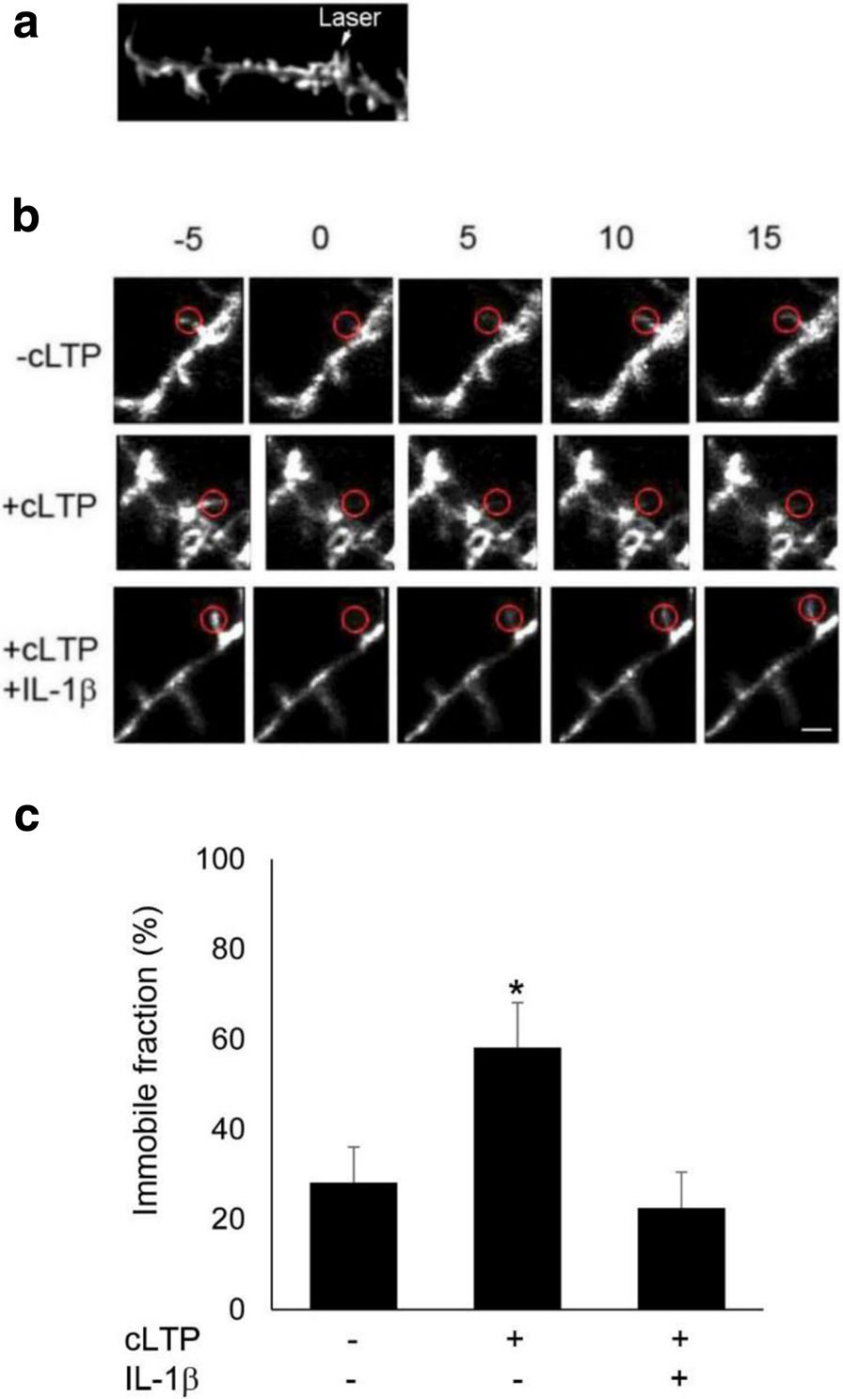
IL-1β treatment decreased the cLTP-induced F-actin stabilization. FRAP analyses were performed using hippocampal neurons at 16–18 DIV that were transfected with Lifeact-GFP. **a** Illustration of FRAP setting. **b** Representative fluorescence images show spines of control group (-cLTP, top panel), cLTP group (middle panel), and cLTP+IL-1β group (bottom) during FRAP. Scale bar, 2 μm **c** Analysis of immobile fractions from data obtained in **b**. The mobile fraction and the immobile fraction (fi) were calculated as described in the “Methods” section. Data are mean ± SEM (*n* = 4, (*p < 0.05, ANOVA)

To explore the mechanism underlying the effect of IL-1β-mediated impairment on actin dynamics, we performed live-cell imaging, using FRAP (fluorescence recovery after photobleaching) assays (Fig. 4a) to monitor the turnover of actin filaments at single spines. This assay is based on the fluorescence recovery of Lifeact-GFP after photobleaching using time-lapse imaging [32]. To analyze the movement of actin, FRAP analyses were conducted after transfection of Lifeact-GFP. The actin cytoskeleton shows a very high turnover rate in dendritic spines. We observed that the recovery of Lifeact-GFP was rapidly completed within 5 min, a time consistent with a previous report [33] (Fig. 4b, top panel). The FRAP assay confirmed that cLTP induces F-actin stabilization (Fig. 4b, middle panel, and c, 25.6 ± 11% of immobile fraction before cLTP vs 62.8 ± 6.4% of immobile fraction 20s after cLTP, *n* = 4), consistent with previous finding [34]. However, cLTP treatment failed to stabilize F-actin in IL-1β-treated neurons (Fig. 4b, bottom panel, and c, 22.5 ± 9% of immobile fraction in IL-1β-treated group vs 58.2 ± 10.2% of immobile fraction in control group, *n* = 4). Under control conditions, IL-1β treatment did not affect significantly actin dynamics (18.6 ± 4.5% of immobile fraction in IL-1β-treated group vs 28.1 ± 8% of immobile fraction in control group, *n* = 4). These results suggest that IL-1β impairs cLTP-induced F-actin stabilization during spine remodeling after cLTP.

### Cofilin signaling contributes to the inhibitory effects of IL-1β on cLTP-induced GluA1 insertion

**Fig. 5:**
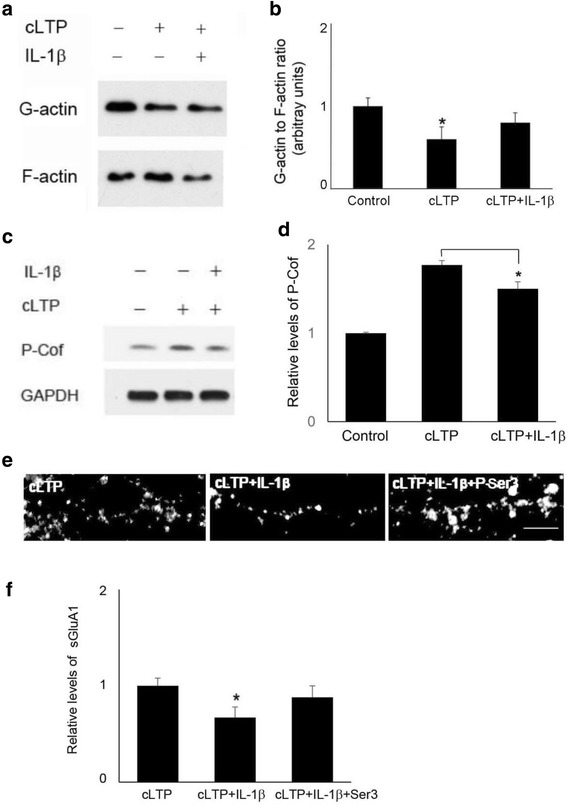
IL-1β impaired cLTP-induced signaling associated with actin dynamics. **a** Western blots of G-actin and F-actin. **b** Quantification of the blots shown in **a**. Data are mean ± SEM from three independent experiments expressed in terms of the ratio of G-actin to F-actin obtained in the control cultures (*p < 0.05, ANOVA). **c** Western blots of phosphorylation of cofilin (P-Cof). **d** Quantification of the blots shown in **c**. Data are means ± SE of values from three independent experiments expressed in terms of P-Cof obtained in the control cultures (*p < 0.05, ANOVA). **e** Representative fluorescence images show the expression of sGluA1 measured by immunostaining in cells treated with cLTP in the presence or absence of IL-1β with or without Ser3 peptide. Scale bar, 5 μm. **f** Quantification of the images shown in **e**. Data are mean ± SEM from three independent experiments expressed in terms of control (*p < 0.05, ANOVA)

To confirm the effect of IL-1β on cLTP-induced actin polymerization, we first measured the ratio of G-actin and F-actin, which is composed of aggregated G-actin. The ratio of F-actin to G-actin, which reflects the balance between actin polymerization and depolymerization, was increased by cLTP and reduced by IL-1β (Fig. 5a, b). Next, we examined the effect of IL-1β on molecules that regulate actin dynamics. Cofilin is an actin-binding protein whose activation depolymerizes actin filaments [35]. Given that theta-LTP increased cofilin phosphorylation at Ser3 [35], which inhibits cofilin activity, and that IL-1β decreased brain-derived neurotrophic factor (BDNF)-induced cofilin phosphorylation in CA1 of organotypic hippocampal slices [35], we reasoned that IL-1β might affect cLTP-induced actin polymerization through modulating cofilin signaling. Indeed, IL-1β treatment decreased cLTP-induced increase in cofilin phosphorylation (177 + 5% of control by cLTP vs 150 + 8% of control by cLTP and IL-1β, *n* = 4). (Fig. 5c, d), indicating that IL-1β impairs cLTP-induced suppression of cofilin activity. We further tested whether an IL-1β-mediated decrease of cLTP-induced GluA1 insertion can be reversed by decreasing cofilin activity. Since cofilin is inactivated by phosphorylation at Ser3, we used a peptide consisting of 1-16 residues of cofilin with Ser3-phosphorylation (P-Ser3) [36] to block endogenous cofilin activity. P-Ser3 treatment prevented IL-1β-mediated decrease of cLTP-induced GluA1 insertion (Fig. 5e, f).

### IL-1β-mediated impairment of cLTP-induced synaptic plasticity requires ceramide signaling

**Fig. 6:**
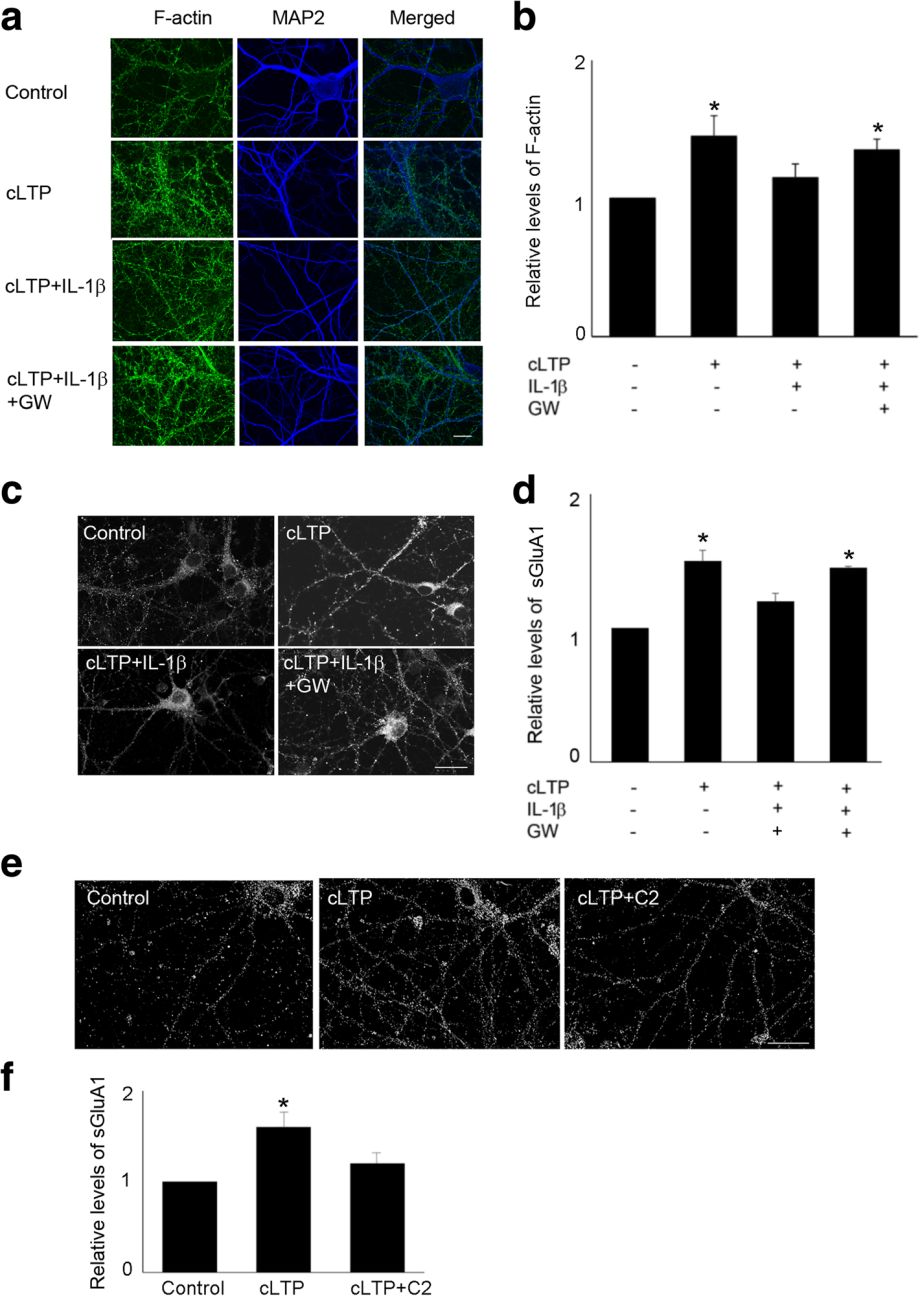
Neutral sphingomyelinase inhibitor GW4869 attenuated IL-1β-mediated suppression of cLTP-induced synaptic plasticity. **a** Representative fluorescence images show F-actin, which was stained by phallodin, in unstimulated control cells and cells treated with cLTP in the presence or absence of IL-1β with or without GW4869 (5 μM). Scale bar, 10 μm. **b** Quantification of the images shown in **a**. The intensity of phallodin staining was normalized by MAP2 staining. Data are mean ± SEM from three independent experiments expressed in terms of control (*p < 0.05, ANOVA). **c** Neutral sphingomyelinase inhibitor GW4869 (5 μM) attenuated IL-1β-mediated suppression of cLTP-induced GluA1 insertion. Representative fluorescence images show the expression of sGluA1 in unstimulated control cells and cells treated with cLTP in the presence or absence of IL-1β with or without GW4869. Scale bar, 10 μm. **d** Quantification of the images shown in **c**. Data are mean ± SEM from three independent experiments expressed in terms of control (*p < 0.05, ANOVA). **e** Representative fluorescence images show the expression of sGluA1 in unstimulated control cells and cells treated with cLTP in the presence or absence of C2-ceramide (10 μM). Scale bar, 20 μm. **f** Quantification of the images shown in **e**. Data are mean ± SEM from three independent experiments expressed in terms of control (*p < 0.05, ANOVA)

Previously, we showed that IL-1β suppresses BDNF neuroprotection via a sphingolipids/ceramide pathway [37]. Sphingolipids are major components of neuronal membranes, where they are particularly enriched [38]. Ceramide, generated from the sphingomyelin pathway, is a major sphingolipid contributing to synaptic plasticity. Indeed, synthetic cell-permeable ceramide analogs increase excitatory postsynaptic currents transiently and led to sustained depression of excitatory postsynaptic currents [39–41]. Further, ceramide mediates cellular signals of cytokines such as tumor necrosis factor-α that are rapidly produced in the brain in response to vigorous neuronal activity and tissue injury [42, 43]. As ceramide generation may occur via either de novo synthesis or the hydrolysis of sphingomylin by acidic or neutral sphingomyelinases, we first examined the effect of the inhibitors of sphingomyelinases, selective to these ceramide-producing routes. Specifically, we tested the effects of GW4869 (a neutral sphingomyelinase inhibitor), desipramine (an acidic sphingomyelinase inhibitor), and fumonisin B1 (FB1) (an inhibitor of the de novo pathway) on IL-1β-mediated impairment of cLTP-induced spine plasticity. Inhibitors of neutral sphingomyelinase significantly attenuated both the IL-1β-mediated suppression of actin polymerization (Fig. 6a, b) and the expression of sGluA1 following cLTP (Fig. 6c, d). In contrast, neither the de novo pathway inhibitor FB1 nor desipramine, an inhibitor of acidic sphingomyelinase, affected IL-1β-mediated suppression of cLTP-induced expression of sGluA1 (data not shown). Moreover, consistent with the idea that IL-1β suppresses spine plasticity via ceramide generation, C2-ceramide treatment decreased cLTP-induced expression of sGluA1 (Fig. 6e, f). These results suggest that sphingomyelin pathway is involved into IL-1β-mediated impairment of cLTP-induced synaptic plasticity.

### Src activation by IL-1β is required for IL-1β-mediated impairment of spine cLTP

**Fig. 7:**
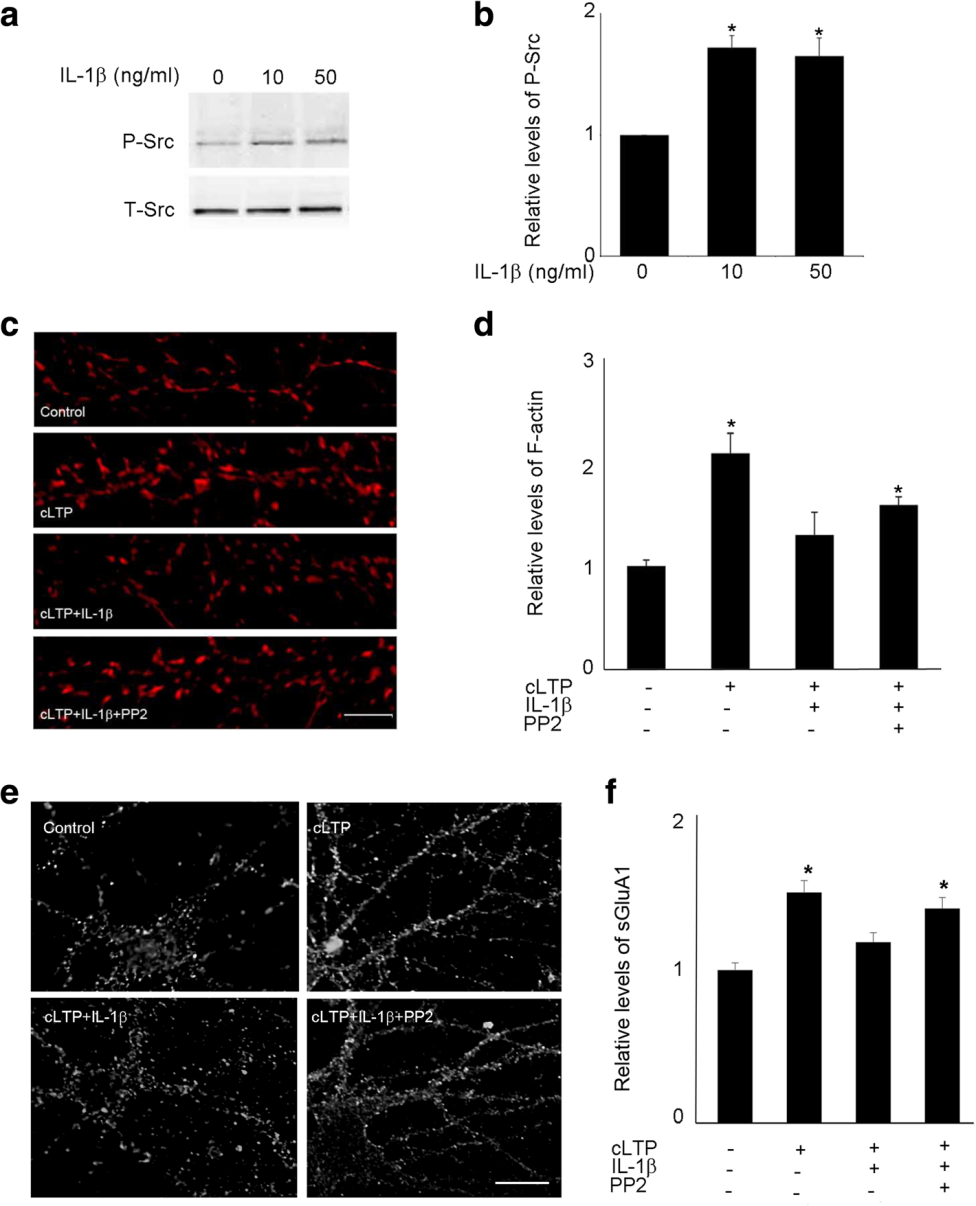
Src inhibitor PP2 attenuated IL-1β-mediated suppression of cLTP-induced synaptic plasticity. **a** Gel image shows Western blots of phosphorylated Src (P-Src). Hippocampal neurons were exposed to IL-1β for 20 min. **b** Quantification of the blots shown in **a.** Data are mean ± SEM from three independent experiments expressed in terms of P-Src obtained in the control cultures (*p < 0.05, ANOVA). **c** Representative fluorescence images show F-actin labeled by phallodin in unstimulated control cells and cells treated with cLTP in the presence or absence of IL-1β with or without PP2 (10 μM). Scale bars, 5 μm. **d** Quantification of the images shown in **c**. Data are mean ± SEM from three independent experiments expressed in terms of control (*p < 0.05, ANOVA). **e** Representative fluorescence images show the expression of sGluA1 in unstimulated control cells and cells treated with cLTP in the presence or absence of IL-1β with or without PP2. Scale bar, 10 μm. **f** Quantification of the images shown in **e**. Data are mean ± SEM from three independent experiments expressed in terms of control (*p < 0.05, ANOVA)

It has been reported that IL-1β [40, 44] and C2-ceramide [45] can activate Src, a protein tyrosine kinase that controls many functions, including cell adhesion, growth, movement, and differentiation [46]*.* Consistent with previous reports, we observed that IL-1β treatment increased Src phosphorylation in cultured hippocampal neurons (Fig. 7a, b). To identify the downstream target of IL-1β-induced ceramide activation, we examined the role of Src in IL-1β-mediated impairment of cLTP-induced synaptic plasticity. Pyrazolopyrimidine 2 (PP2), a Src inhibitor, decreased IL-1β-induced suppression of cLTP-induced actin polymerization (Fig. 7c, d). Pretreatment with PP2 also prevented IL-1β-induced impairment of cLTP-induced GluA1 insertion (Fig. 7e, f). These results suggest that activation of Src via ceramide contributes to the suppressive effect of IL-1β on cLTP-induced synaptic plasticity.

## Discussion

In this study, we demonstrated that IL-1β impairs cLTP-induced AMPA receptor insertion and spine growth in cultured hippocampal neurons shortly after stimulation by interfering with actin dynamics through a ceramide-associated mechanism.

To focus on neuron-specific effects, we used a cell culture model of chemical LTP that uses glycine stimulation to selectively activate synaptic NMDA receptors in cultured neurons [24]. It is noteworthy that several lines of evidence have demonstrate that glycine-induced cLTP and the classic electrophysiological theta-burst-induced LTP (theta-LTP) share cellular processes that are key to activity-dependent plasticity [23]. cLTP and theta-LTP share the key features of LTP including NMDA receptor-dependent activation of CAMKII [22], AMPA receptor insertion to the postsynaptic membrane, and increase in dendritic spine volume [25, 28, 47]. While proinflammatory cytokines such as IL-1β [48] and TNFα [49] have been shown to impair activity-dependent signaling and survival, the effect of cytokines on cLTP-induced synaptic plasticity in primary neuronal culture has not been studied. Our data disclose a cascade by which IL-1β, through activating N-SMase and increasing Src activity, affects activity-dependent synaptic remodeling in spines. This cascade, which conveys IL-1β signal from membrane to actin cytoskeleton and AMPA receptor insertion, may represent an underlying mechanism that contributes to IL-1β-dependent inflammation-induced LTP deficits in in vivo models of AD where glia activation increases IL-1β [1, 11].

In our study, we showed that the effect of IL-1β on cLTP-induced AMPA receptor insertion and spine enlargement correlates with the inhibitory effect of IL-1β on cLTP-induced F-actin formation and stabilization in spines and can be prevented by a peptide that stabilizes actin filaments. Actin is highly enriched in dendritic spines and provides the foundation for the structural changes accompanying LTP, including an increased number of spines [29, 47, 50], and supports the scaffold for anchoring and clustering glutamate receptors [29, 51]. Actin depolymerization by latrunculin elicits AMPA receptor internalization [52]. By contrast, stabilization of F-actin by jasplakinolide prevents AMPA receptor endocytosis. Actin polymerization is required for the production of stable LTP after theta-burst stimulation. In the theta-LTP paradigm, two synaptic signaling cascades for LTP induction have been proposed: RhoA-ROCK-cofilin leads to actin polymerization, whereas Rac-PAK stabilizes the newly formed filaments [35]. Our data that IL-1β suppresses F-actin formation, GluA1 surface insertion, and cofilin deactivation (phosphorylation) during early phase cLTP in cultured neurons, including live-cell imaging, support a model based on direct IL-1β action on the dendritic spine. Also supporting the local and neuron-specific deleterious action of IL-1β on LTP, we have found that this cytokine directly suppresses cLTP in isolated hippocampal synaptosomes, which contain IL-1 receptor subunits [26].

We investigated the role of sphingolipids in IL-1β-mediated impairment of cLTP-induced synaptic plasticity. Our data indicate that the IL-1β-induced impairment of cLTP is mediated, at least in part, by activation of neutral sphingomyelinase via Src signaling. Our study identified a role of Src activation in the molecular mechanism by which IL-1β locally affects cLTP-induced actin remodeling and AMPA receptor insertion. Our data are consistent with previous finding that IL-1β can initiate a signaling pathway that activates Src family kinases (SFKs) to modulate Ca^2+^ signaling in central neurons. IL-1β-mediated activation of SFKs enhances Ca^2+^ influx via NMDA receptors [44]. IL-1β activates neutral sphingomyelinase to produce ceramide, which subsequently activates Src kinase in hypothalamic neurons [40, 53, 54] and hippocampal neurons [55]. The IL-1β-mediated activation of neutral sphingomyelinase depends on MyD88 [40], an adaptor protein that we found is essential for the suppression of cLTP by IL-1β in aged synaptosomes [26].

Importantly, Src is closely associated with the regulation of actin cytoskeleton. The Src-mediated regulation of F-actin dynamics has been examined in neuronal migration [56]. In this paradigm, overexpression of Src induced dephosphorylation of cofilin as Ser3. The connection between Src and actin dynamics also has been indicated in Chorea-acanthocytosis, a neurodegenerative disease [57]. In disease-specific-induced pluripotent stem cells (iPSCs), the pathologically altered synaptic activity in ChAc neurons was reversed by F-actin stabilizer phallacidin and the Src kinase inhibitor PP2 [57]. The influence of hyperactive Src on synaptic plasticity has been demonstrated in p140Cap knock-out mice [58]. p140Cap, a scaffold protein, localizes into dendritic spines and interacts with Src as an inhibitor [58]. p140Cap−/− mice display specific learning defects, F-actin disorganization, and impaired LTP. In this model, activated Src inhibits actin polymerization through phosphorylation of the RhoA-specific GTPase-activating protein p190RhoGAP and interacting with RhoA-binding protein Citron-N. The loss of p140Cap results in hyperactivation of Src kinases and leads to a decrease in actin polymerization through downregulation of RhoA/ROCK/cofilin pathway. In addition, the defects in synaptic plasticity of p140Cap−/− primary neurons can be reverted by Src inhibitors. Consistent with the model that hyperactive Src impairs RhoA/ROCK/cofilin pathway, we also found that IL-1β decreased cofilin phosphorylation and F-actin levels after cLTP. As BDNF also activates RhoA/ROCK/cofilin pathway, we cannot, however, rule out the contribution of possible IL-1β-mediated suppression of endogenous BDNF signaling during cLTP to IL-1β effect on synaptic plasticity. Our data provide another link between IL-1β signaling and actin polymerization. Thus, multiple mechanisms that link IL-1β signaling to RhoA and actin cytoskeletal dynamics may act collectively and contribute to inflammation-induced declines in learning and memory functions.

## Conclusion

By focusing on the structural changes during cLTP, our study identified a critical link between ceramide-mediated Src activation, a well-documented signaling pathway of IL-1β, and actin dynamics, thus providing insights into the mechanism underlying inflammation-mediated impairments of learning and memory.

## References

[1] Heneka MT, Kummer MP, Latz E (2014). Innate immune activation in neurodegenerative disease. Nat Rev Immunol.

[2] Heppner FL, Ransohoff RM, Becher B (2015). Immune attack: the role of inflammation in Alzheimer disease. Nat Rev Neurosci.

[3] Shaftel SS, Griffin WS, O'Banion MK (2008). The role of interleukin-1 in neuroinflammation and Alzheimer disease: an evolving perspective. J Neuroinflammation.

[4] Cotman CW, Hailer NP, Pfister KK, Soltesz I, Schachner M (1998). Cell adhesion molecules in neural plasticity and pathology: similar mechanisms, distinct organizations?. Prog Neurobiol.

[5] Rachal Pugh C, Fleshner M, Watkins LR, Maier SF, Rudy JW (2001). The immune system and memory consolidation: a role for the cytokine IL-1beta. Neurosci Biobehav Rev.

[6] Hein AM, Stasko MR, Matousek SB, Scott-McKean JJ, Maier SF, Olschowka JA, Costa AC, O'Banion MK (2010). Sustained hippocampal IL-1beta overexpression impairs contextual and spatial memory in transgenic mice. Brain Behav Immun.

[7] Barrientos RM, Frank MG, Hein AM, Higgins EA, Watkins LR, Rudy JW, Maier SF (2009). Time course of hippocampal IL-1 beta and memory consolidation impairments in aging rats following peripheral infection. Brain Behav Immun.

[8] Frank MG, Barrientos RM, Hein AM, Biedenkapp JC, Watkins LR, Maier SF (2010). IL-1RA blocks E. coli-induced suppression of Arc and long-term memory in aged F344xBN F1 rats. Brain Behav Immun.

[9] Bellinger FP, Madamba S, Siggins GR (1993). Interleukin 1 beta inhibits synaptic strength and long-term potentiation in the rat CA1 hippocampus. Brain Res.

[10] Ross FM, Allan SM, Rothwell NJ, Verkhratsky A (2003). A dual role for interleukin-1 in LTP in mouse hippocampal slices. J Neuroimmunol.

[11] Lynch MA (2015). Neuroinflammatory changes negatively impact on LTP: a focus on IL-1beta. Brain Res.

[12] Otto C, Kovalchuk Y, Wolfer DP, Gass P, Martin M, Zuschratter W, Grone HJ, Kellendonk C, Tronche F, Maldonado R (2001). Impairment of mossy fiber long-term potentiation and associative learning in pituitary adenylate cyclase activating polypeptide type I receptor-deficient mice. J Neurosci.

[13] Chapman TR, Barrientos RM, Ahrendsen JT, Maier SF, Patterson SL (2010). Synaptic correlates of increased cognitive vulnerability with aging: peripheral immune challenge and aging interact to disrupt theta-burst late-phase long-term potentiation in hippocampal area CA1. J Neurosci.

[14] Gallagher JJ, Finnegan ME, Grehan B, Dobson J, Collingwood JF, Lynch MA (2012). Modest amyloid deposition is associated with iron dysregulation, microglial activation, and oxidative stress. J Alzheimers Dis.

[15] Gallagher JJ, Minogue AM, Lynch MA (2013). Impaired performance of female APP/PS1 mice in the Morris water maze is coupled with increased Abeta accumulation and microglial activation. Neurodegener Dis.

[16] Kitazawa M, Cheng D, Tsukamoto MR, Koike MA, Wes PD, Vasilevko V, Cribbs DH, LaFerla FM (2011). Blocking IL-1 signaling rescues cognition, attenuates tau pathology, and restores neuronal beta-catenin pathway function in an Alzheimer's disease model. J Immunol.

[17] Sala C, Segal M (2014). Dendritic spines: the locus of structural and functional plasticity. Physiol Rev.

[18] Oh MC, Derkach VA, Guire ES, Soderling TR (2006). Extrasynaptic membrane trafficking regulated by GluR1 serine 845 phosphorylation primes AMPA receptors for long-term potentiation. J Biol Chem.

[19] Shepherd JD, Huganir RL (2007). The cell biology of synaptic plasticity: AMPA receptor trafficking. Annu Rev Cell Dev Biol.

[20] Hotulainen P, Hoogenraad CC (2010). Actin in dendritic spines: connecting dynamics to function. J Cell Biol.

[21] Tong L, Prieto GA, Kramar EA, Smith ED, Cribbs DH, Lynch G, Cotman CW (2012). Brain-derived neurotrophic factor-dependent synaptic plasticity is suppressed by interleukin-1beta via p38 mitogen-activated protein kinase. J Neurosci.

[22] Molnar E (2011). Long-term potentiation in cultured hippocampal neurons. Semin Cell Dev Biol.

[23] Musleh W, Bi X, Tocco G, Yaghoubi S, Baudry M (1997). Glycine-induced long-term potentiation is associated with structural and functional modifications of alpha-amino-3-hydroxyl-5-methyl-4-isoxazolepropionic acid receptors. Proc Natl Acad Sci U S A.

[24] Lu W, Man H, Ju W, Trimble WS, MacDonald JF, Wang YT (2001). Activation of synaptic NMDA receptors induces membrane insertion of new AMPA receptors and LTP in cultured hippocampal neurons. Neuron.

[25] Fortin DA, Davare MA, Srivastava T, Brady JD, Nygaard S, Derkach VA, Soderling TR (2010). Long-term potentiation-dependent spine enlargement requires synaptic Ca2+-permeable AMPA receptors recruited by CaM-kinase I. J Neurosci.

[26] Prieto GA, Snigdha S, Baglietto-Vargas D, Smith ED, Berchtold NC, Tong L, Ajami D, LaFerla FM, Rebek J, Cotman CW (2015). Synapse-specific IL-1 receptor subunit reconfiguration augments vulnerability to IL-1beta in the aged hippocampus. Proc Natl Acad Sci U S A.

[27] Kennedy MJ, Davison IG, Robinson CG, Ehlers MD (2010). Syntaxin-4 defines a domain for activity-dependent exocytosis in dendritic spines. Cell.

[28] Park M, Penick EC, Edwards JG, Kauer JA, Ehlers MD (2004). Recycling endosomes supply AMPA receptors for LTP. Science.

[29] Park M, Salgado JM, Ostroff L, Helton TD, Robinson CG, Harris KM, Ehlers MD (2006). Plasticity-induced growth of dendritic spines by exocytic trafficking from recycling endosomes. Neuron.

[30] Krucker T, Siggins GR, Halpain S (2000). Dynamic actin filaments are required for stable long-term potentiation (LTP) in area CA1 of the hippocampus. Proc Natl Acad Sci U S A.

[31] Riedl J, Crevenna AH, Kessenbrock K, Yu JH, Neukirchen D, Bista M, Bradke F, Jenne D, Holak TA, Werb Z (2008). Lifeact: a versatile marker to visualize F-actin. Nat Methods.

[32] Rocca DL, Amici M, Antoniou A, Blanco Suarez E, Halemani N, Murk K, McGarvey J, Jaafari N, Mellor JR, Collingridge GL, Hanley JG (2013). The small GTPase Arf1 modulates Arp2/3-mediated actin polymerization via PICK1 to regulate synaptic plasticity. Neuron.

[33] Bae J, Sung BH, Cho IH, Song WK (2012). F-actin-dependent regulation of NESH dynamics in rat hippocampal neurons. PLoS One.

[34] Tanokashira D, Morita T, Hayashi K, Mayanagi T, Fukumoto K, Kubota Y, Yamashita T, Sobue K (2012). Glucocorticoid suppresses dendritic spine development mediated by down-regulation of caldesmon expression. J Neurosci.

[35] Rex CS, Chen LY, Sharma A, Liu J, Babayan AH, Gall CM, Lynch G (2009). Different Rho GTPase-dependent signaling pathways initiate sequential steps in the consolidation of long-term potentiation. J Cell Biol.

[36] Zhou Q, Homma KJ, Poo MM (2004). Shrinkage of dendritic spines associated with long-term depression of hippocampal synapses. Neuron.

[37] Tong L, Balazs R, Soiampornkul R, Thangnipon W, Cotman CW. Interleukin-1 beta impairs brain derived neurotrophic factor-induced signal transduction. Neurobiol Aging. 2008:29:1380–393. 10.1016/j.neurobiolaging.2007.02.027 PMC4052889 17467122

[38] Schwarz A, Rapaport E, Hirschberg K, Futerman AH (1995). A regulatory role for sphingolipids in neuronal growth. Inhibition of sphingolipid synthesis and degradation have opposite effects on axonal branching. J Biol Chem.

[39] Coogan AN, O'Neill LA, O'Connor JJ (1999). The P38 mitogen-activated protein kinase inhibitor SB203580 antagonizes the inhibitory effects of interleukin-1beta on long-term potentiation in the rat dentate gyrus in vitro. Neuroscience.

[40] Davis CN, Tabarean I, Gaidarova S, Behrens MM, Bartfai T (2006). IL-1beta induces a MyD88-dependent and ceramide-mediated activation of Src in anterior hypothalamic neurons. J Neurochem.

[41] Yang SN (2000). Ceramide-induced sustained depression of synaptic currents mediated by ionotropic glutamate receptors in the hippocampus: an essential role of postsynaptic protein phosphatases. Neuroscience.

[42] Kim MY, Linardic C, Obeid L, Hannun Y (1991). Identification of sphingomyelin turnover as an effector mechanism for the action of tumor necrosis factor alpha and gamma-interferon. Specific role in cell differentiation. J Biol Chem.

[43] Tchelingerian JL, Quinonero J, Booss J, Jacque C (1993). Localization of TNF alpha and IL-1 alpha immunoreactivities in striatal neurons after surgical injury to the hippocampus. Neuron.

[44] Viviani B, Bartesaghi S, Gardoni F, Vezzani A, Behrens MM, Bartfai T, Binaglia M, Corsini E, Di Luca M, Galli CL, Marinovich M (2003). Interleukin-1beta enhances NMDA receptor-mediated intracellular calcium increase through activation of the Src family of kinases. J Neurosci.

[45] Ibitayo AI, Tsunoda Y, Nozu F, Owyang C, Bitar KN (1998). Src kinase and PI 3-kinase as a transduction pathway in ceramide-induced contraction of colonic smooth muscle. Am J Phys.

[46] Thomas SM, Brugge JS (1997). Cellular functions regulated by Src family kinases. Annu Rev Cell Dev Biol.

[47] Kopec C, Malinow R (2006). Neuroscience. Matters of size. Science.

[48] Smith ED, Prieto GA, Tong L, Sears-Kraxberger I, Rice JD, Steward O, Cotman CW (2014). Rapamycin and interleukin-1beta impair brain-derived neurotrophic factor-dependent neuron survival by modulating autophagy. J Biol Chem.

[49] Zhao X, Bausano B, Pike BR, Newcomb-Fernandez JK, Wang KK, Shohami E, Ringger NC, DeFord SM, Anderson DK, Hayes RL (2001). TNF-alpha stimulates caspase-3 activation and apoptotic cell death in primary septo-hippocampal cultures. J Neurosci Res.

[50] Fedulov V, Rex CS, Simmons DA, Palmer L, Gall CM, Lynch G (2007). Evidence that long-term potentiation occurs within individual hippocampal synapses during learning. J Neurosci.

[51] Matsuzaki M, Honkura N, Ellis-Davies GC, Kasai H (2004). Structural basis of long-term potentiation in single dendritic spines. Nature.

[52] Zhou Q, Xiao M, Nicoll RA (2001). Contribution of cytoskeleton to the internalization of AMPA receptors. Proc Natl Acad Sci U S A.

[53] Davis CN, Mann E, Behrens MM, Gaidarova S, Rebek M, Rebek J, Bartfai T (2006). MyD88-dependent and -independent signaling by IL-1 in neurons probed by bifunctional Toll/IL-1 receptor domain/BB-loop mimetics. Proc Natl Acad Sci U S A.

[54] Sanchez-Alavez M, Tabarean IV, Behrens MM, Bartfai T (2006). Ceramide mediates the rapid phase of febrile response to IL-1beta. Proc Natl Acad Sci U S A.

[55] Ghosh B, Green MV, Krogh KA, Thayer SA (2016). Interleukin-1beta activates an Src family kinase to stimulate the plasma membrane Ca2+ pump in hippocampal neurons. J Neurophysiol.

[56] Wang JT, Song LZ, Li LL, Zhang W, Chai XJ, An L, Chen SL, Frotscher M, Zhao ST (2015). Src controls neuronal migration by regulating the activity of FAK and cofilin. Neuroscience.

[57] Stanslowsky N, Reinhardt P, Glass H, Kalmbach N, Naujock M, Hensel N, Lubben V, Pal A, Venneri A, Lupo F (2016). Neuronal dysfunction in iPSC-derived medium spiny neurons from Chorea-Acanthocytosis patients is reversed by Src kinase inhibition and F-actin stabilization. J Neurosci.

[58] Repetto D, Camera P, Melani R, Morello N, Russo I, Calcagno E, Tomasoni R, Bianchi F, Berto G, Giustetto M (2014). p140Cap regulates memory and synaptic plasticity through Src-mediated and citron-N-mediated actin reorganization. J Neurosci.

